# Recent progress in silk fibroin-based flexible electronics

**DOI:** 10.1038/s41378-021-00261-2

**Published:** 2021-05-06

**Authors:** Dan-Liang Wen, De-Heng Sun, Peng Huang, Wen Huang, Meng Su, Ya Wang, Meng-Di Han, Beomjoon Kim, Juergen Brugger, Hai-Xia Zhang, Xiao-Sheng Zhang

**Affiliations:** 1 School of Electronic Science and Engineering, University of Electronic Science and Technology of China, Chengdu, 611731 China; 2 CIRMM, Institute of Industrial Science, The University of Tokyo, Tokyo, 153-8505 Japan; 3 Microsystems Laboratory, École Polytechnique Fédérale de Lausanne (EPFL), 1015 Lausanne, Switzerland; 4 Institute of Microelectronics, Peking University, 100087 Beijing, China

**Keywords:** Electrical and electronic engineering, Electronic properties and materials

## Abstract

With the rapid development of the Internet of Things (IoT) and the emergence of 5G, traditional silicon-based electronics no longer fully meet market demands such as nonplanar application scenarios due to mechanical mismatch. This provides unprecedented opportunities for flexible electronics that bypass the physical rigidity through the introduction of flexible materials. In recent decades, biological materials with outstanding biocompatibility and biodegradability, which are considered some of the most promising candidates for next-generation flexible electronics, have received increasing attention, e.g., silk fibroin, cellulose, pectin, chitosan, and melanin. Among them, silk fibroin presents greater superiorities in biocompatibility and biodegradability, and moreover, it also possesses a variety of attractive properties, such as adjustable water solubility, remarkable optical transmittance, high mechanical robustness, light weight, and ease of processing, which are partially or even completely lacking in other biological materials. Therefore, silk fibroin has been widely used as fundamental components for the construction of biocompatible flexible electronics, particularly for wearable and implantable devices. Furthermore, in recent years, more attention has been paid to the investigation of the functional characteristics of silk fibroin, such as the dielectric properties, piezoelectric properties, strong ability to lose electrons, and sensitivity to environmental variables. Here, this paper not only reviews the preparation technologies for various forms of silk fibroin and the recent progress in the use of silk fibroin as a fundamental material but also focuses on the recent advanced works in which silk fibroin serves as functional components. Additionally, the challenges and future development of silk fibroin-based flexible electronics are summarized.

**Figure:**
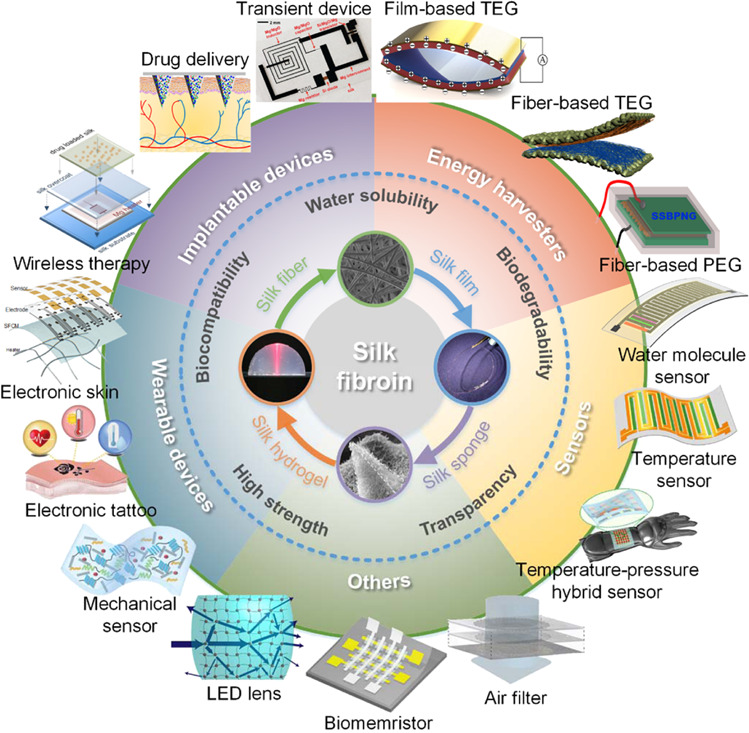
(1) This review focuses on silk fibroin serving as active functional components to construct flexible electronics. (2) Recent representative reports on flexible electronic devices that applied silk fibroin as fundamental supporting components are summarized. (3) This review summarizes the current typical silk fibroin-based materials and the corresponding advanced preparation technologies. (4) The current challenges and future development of silk fibroin-based flexible electronic devices are analyzed.

(1) This review focuses on silk fibroin serving as active functional components to construct flexible electronics. (2) Recent representative reports on flexible electronic devices that applied silk fibroin as fundamental supporting components are summarized. (3) This review summarizes the current typical silk fibroin-based materials and the corresponding advanced preparation technologies. (4) The current challenges and future development of silk fibroin-based flexible electronic devices are analyzed.

## Introduction

In the past decade, the rapid development of flexible electronics has been witnessed through the surge in the market and the emergence of diverse devices, including flexible sensors/actuators^1–4^, flexible cells^5,6^, flexible displays^7,8^, electronic skins^9,10^, flexible integrated microsystems^11,12^, as well as the comprehensive coverage of application fields, involving information^13,14^, energy^15,16^, healthcare^17,18^, and national defense^19,20^. Thus, flexible electronics have made a certain impact on almost all aspects of the daily life of human beings. This is an inevitable result of flexible electronic devices overcoming the physical rigidity of traditional silicon-based electronics and well solving the mechanical mismatch between electronic devices and nonplanar surfaces in most application scenarios. The global market of commercial flexible electronics is expected to generate a revenue of $77.3 billion by the end of 2029, growing at a compound annual growth rate of 8.5% between 2018 and 2029^21^. However, at present, the further development of flexible electronics is confronted with many essential challenges, for instance, superior flexible materials, sustainable energy supply, and multifunctional integration. In general, a flexible electronic device is realized by preparing functional components made of organic/inorganic materials on a flexible substrate, which endows the device with excellent bendability, stretchability, and adaptability while ensuring normal working conditions. Flexible materials, including the most widely used materials of polytetrafluoroethylene^22^, polydimethylsiloxane (PDMS)^23^, polyimide^24^, fluorinated ethylene propylene^25^, and silicone rubber^26^, play a crucial role in achieving flexibility of electronic devices due to their remarkable mechanical characteristics of long-term repeatable bendability and/or stretchability. However, most of them can hardly achieve the desired biocompatibility and biodegradability, which are essential for wearable and implantable applications. Additionally, most of these materials are expensive, leading to obstacles in large-scale applications.

To address the above issues, researchers have made many attempts to develop flexible natural biomaterials, e.g., cellulose, pectin, chitosan, melanin, and silk fibroin (SF), that are biocompatible, biodegradable, and even economical^27–30^. Meanwhile, biological materials have been extracted from organisms, enabling them to be sustainably produced on a large scale. Therefore, biological materials are some of the most promising candidate materials for next-generation flexible electronic devices. Table S1 in the Supporting Information file compares the characteristics of these flexible natural biomaterials. Among them, cellulose accounts for nearly 40% of the weight of wood, so it is the most abundant natural material on earth^31^. In general, a cellulose solution is obtained from plant cell walls by using mechanical or chemical methods, which can be subsequently processed (e.g., squeezed or doped) and then dried to form cellulose-based functional materials^32^. With the help of the excellent mechanical properties, appealing electrochemical properties, low cost, and simple fabrication, cellulose, and cellulose-based materials (i.e., cellulose fiber and paper) have been widely implemented to construct flexible electronic devices, serving as fundamental supporting components and functional components, including organic light-emitting diodes (OLEDs), organic field-effect transistors (OFETs), and solar cells^33,34^, but their biocompatibility and biodegradability are not excellent. Pectin is also a natural biomaterial extracted from plant cell walls and has also been applied to build flexible electronic devices^35–37^. Due to its good shape memory and excellent permeability to water molecules as well as fast metal ion transport, pectin is more suitable for memory switching devices and sensing devices, but pectin-based flexible materials usually present poor mechanical properties. Chitin and its derivative chitosan have similar molecular structures to cellulose, but they exhibit better biological functions, i.e., biocompatibility and biodegradability^38^. Moreover, chitosan and melanin films have the characteristics of high mechanical strength and good processability; thus, they are a good choice for preparing flexible natural biomaterials^39,40^.

**Fig. 1:**
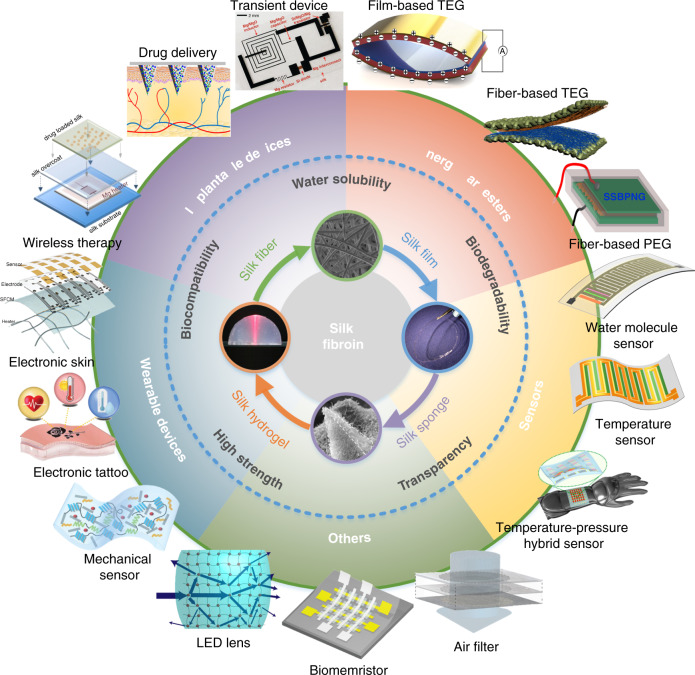
Silk fibroin (SF)-based flexible materials and flexible electronics. Silk fibroin has many superior properties, including remarkable biocompatibility, adjustable biodegradability and water solubility, excellent optical transmittance, and good mechanical strength, and moreover, it can be developed into many forms, e.g., silk fibers, silk films, silk sponges, and silk hydrogels. Therefore, silk fibroin is widely used as fundamental components to construct biocompatible wearable/implantable electronic devices and functional components to form energy harvesters, sensors, filters, lenses, biomemristors, etc.^47–62^. Reproduced with permission from Elsevier (2016), (2018), (2019), ACS (2017), (2019), PNAS (2014), (2017), Wiley (2017), (2019), (2020), AAAS (2012), and Springer Nature (2017), (2020)

SF is extracted from natural silk fibers that are the product of insects, e.g., silkworms and spiders. Both silkworm (*Bombyx mori*) silk and spider silk are composed of glycine-rich proteins, and they have similar structures but different compositions^29^. Spider silk has higher strength and better extensibility^29^; however, preparing SF from silkworm (*Bombyx mori*) cocoons has higher feasibility and practicability because it is conducive to large-scale production^41^. Natural *Bombyx mori* cocoons mainly contain two proteins, SF, which accounts for 70–80 wt% of a cocoon, and sericin, which accounts for 25–30 wt% of a cocoon. It is noteworthy that sericin is usually removed during the preparation process, as it can cause immune responses^42^. SF is composed of α-helices, β-sheet crystals, and random coils, which are assembled by repetitive amino acid sequences with the help of hydrogen bonds, hydrophobic interactions, and van der Waals forces^27^. Compared to the above natural biomaterials, SF possesses greater superiority in biocompatibility and water solubility, and its biodegradability is programmable from hours to years by altering the processing methods or implementing postprocessing procedures (e.g., water vapor annealing, methanol annealing, and ethanol annealing) to change the content of β-sheet crystals and the degree of organization of noncrystalline domains. It has been confirmed that the degradation rate of SF increases with decreasing the content of β-sheet crystals^43,44^. Moreover, β-sheet crystals endow SF-based materials with superior mechanical properties (i.e., high strength and good toughness)^45^. Additionally, SF also demonstrates a unique advantage in solving the issues of a sustainable power supply and multifunctional integration of flexible electronics due to its diverse functional properties. In summary, the superiorities mentioned above coupled with the light weight and transparency as well as ease of processing make the advanced engineering material of SF open new an avenue in the realm of flexible electronics^46^. Figure 1 illustrates the main characteristics and main application forms of SF as well as some typical works on SF-based flexible electronic devices^47–62^.

This review begins with the current classic SF-based materials (i.e., SF solutions, silk fibers, silk films, 3D porous silk sponges, and silk hydrogels) and the corresponding most advanced preparation technologies. Subsequently, we summarize recent typical reports on SF serving as fundamental supporting components, including in wearable flexible electronics and implantable electronics. More importantly, we focus on advanced flexible devices in which SF works as crucial functional components by utilizing its diverse functional properties, e.g., energy harvesters, sensors, filters, biomemristors, and actuators. Finally, we discuss the challenges and future development of SF-based flexible electronic devices.

## Preparation of silk fibroin-based materials

### Preparation of silk fibroin solutions

**Fig. 2:**
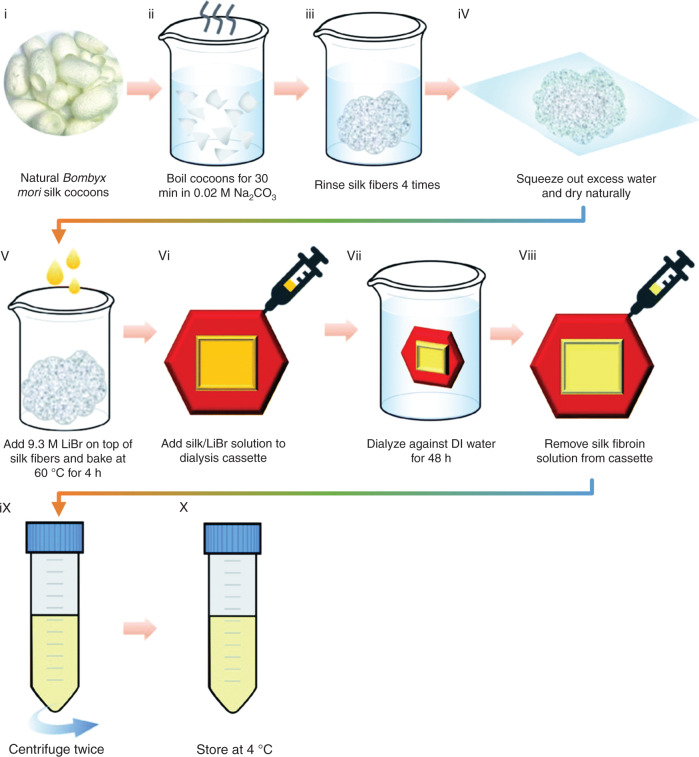
Process flowchart for the regenerated silk fibroin solution prepared from natural *Bombyx mori* cocoons. In addition to the desired silk fibroin, the natural *Bombyx mori* cocoons also mainly contain another protein, sericin, which is the cause of the immune responses and needs to be removed. Subsequently, the extracted silk fibers are prepared in solution form and purified to obtain the regenerated silk fibroin solution. The regenerated silk fibroin solution can be further processed into different shapes, such as silk fibers, silk films, silk sponges, and silk hydrogels, and it can also be doped with other materials to form composite functional materials^63^. Reproduced with permission from RSC (2019)

Most natural biomaterials, including SF, have a common superiority that they can be regenerated, which allows them to be prepared in different shapes (e.g., fibers, films, and porous structures) and doped with other materials to form hybrid functional materials. A SF aqueous solution has outstanding processability, which is obtained after removing harmful sericin from natural *Bombyx mori* cocoons and undergoing a regeneration process, as shown in Fig. 2^63^.

The preparation process of a SF solution can be summarized into four main steps. First, silk fibers are extracted from cocoons, as illustrated in Fig. 2(i)–(iv). The specifics are as follows: (1) an appropriate amount of natural *Bombyx mori* cocoons is cut into small pieces for better dispersion, which is conducive to subsequent sericin removal. (2) A 0.02 M sodium carbonate (Na_2_CO_3_) solution is prepared, and the cocoon pieces are added to the boiling Na_2_CO_3_ solution and boiled for 30–45 min to remove the sericin. Typically, 5 g natural *Bombyx mori* cocoons requires ~2 L Na_2_CO_3_ solution. (3) The extracted silk fibers are rinsed in deionized (DI) water more than four times to remove sericin residue. (4) The dried silk fibers are obtained by squeezing out excess water with tweezers and drying them naturally or in a fume hood. Second, a 9.3 M lithium bromide (LiBr) solution is prepared and added on top of the silk fibers in a beaker, and then, they are placed in a 60 °C oven for 4 h to completely dissolve the silk fibers, as illustrated in Fig. 2(v). Third, the silk/LiBr mixture solution is injected into a 3.5 K MWCO dialysis cassette (or dialysis film) through a syringe and dialyzed against DI water for 48 h to remove LiBr ions and preliminarily obtain a SF solution, as illustrated in Fig. 2(vi), (vii). It is noteworthy that to avoid rupture resulting from water absorption, the dialysis cassette should have a large margin, and moreover, the DI water needs to be substituted five times during the dialysis process. Finally, the preliminary SF solution is transferred from the dialysis cassette into centrifuge tubes with another syringe and centrifuged twice at 9000 rpm for 20 min to remove impurities, as illustrated in Fig. 2(viii), (ix). If necessary, the SF solution can be further purified three times through 5-μm microfiltration. In addition, an environment below 4 °C is usually applied when storing the prepared pure SF solution to decrease the gelation effect^57^. In addition, to expand the functions of silk materials in some cases, with the help of its desired processability, the SF solution is doped with other materials to form composite material solutions before being prepared into solid silk materials, such as gold (Au)-doped SF for surface-enhanced Raman scattering^64^ and others^65,66^. Furthermore, bio-doping methods, such as feeding silkworms with fluorescent dyes, have also been developed to produce environmentally friendly silk fluorescent materials, which is considered as a green route^67,68^.

### Preparation of regenerated silk fibers

**Fig. 3:**
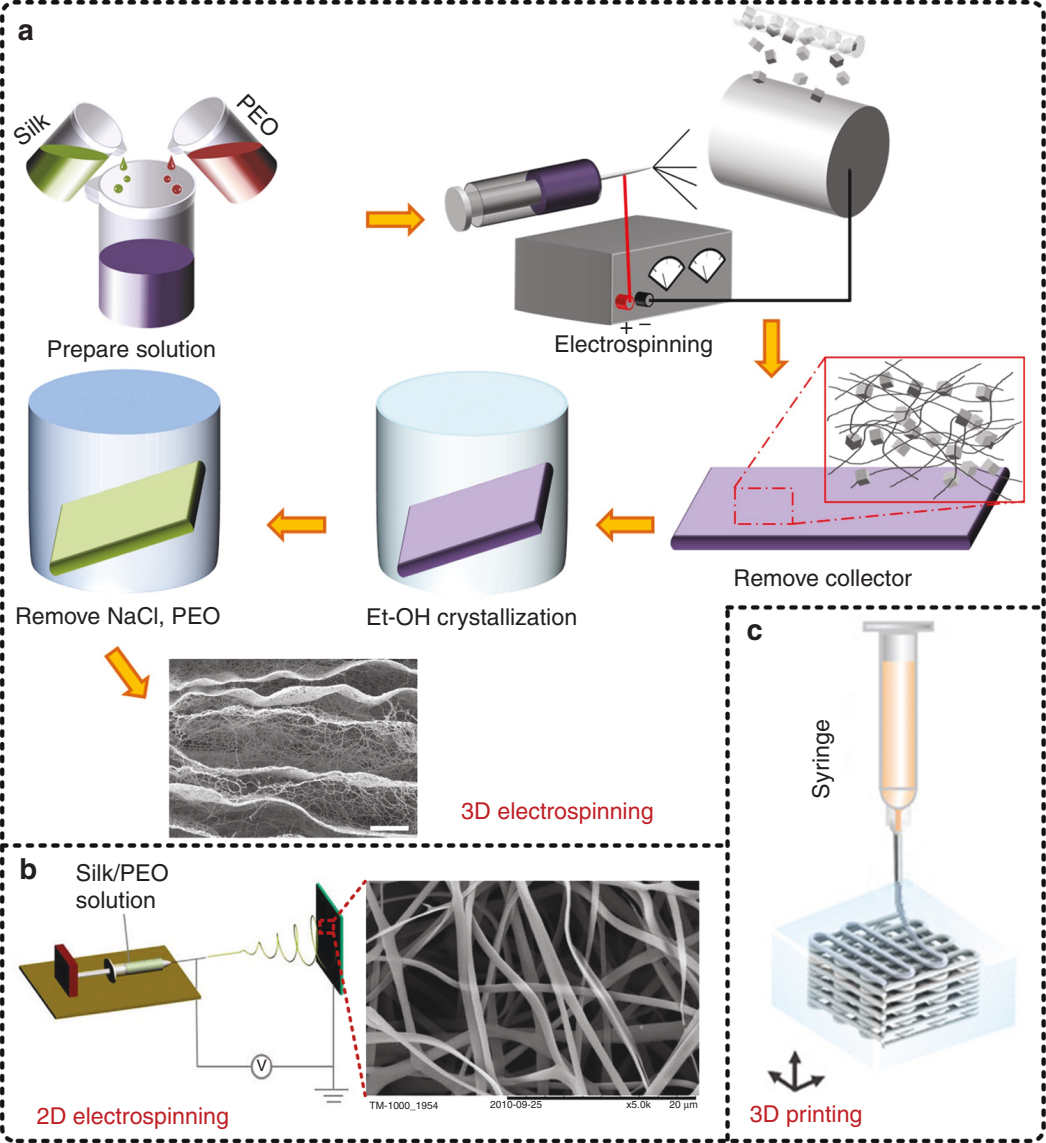
Preparation methods of regenerated silk fibers. **a** Process flowchart for preparing regenerated silk fibers by using 3D electrospinning technology^76^. Reproduced with permission from Elsevier (2016). **b** Schematic diagram of 2D electrospinning technology for regenerated silk fibers^77^. Reproduced with permission from Elsevier (2014). **c** Schematic diagram of 3D printing technology for regenerated silk fibers^78^. Reproduced with permission from Wiley (2019)

In recent years, benefiting from the remarkable mechanical properties in terms of strength and toughness, excellent surface adaptability, and desired air permeability, textile materials based on fiber weaving have attracted increasing attention and made a great contribution in promoting the development of flexible electronic devices. Nanotextiles made of regenerated silk fibers have also been studied in depth^69–72^. Electrospinning, a method for fiber preparation working under a strong electric field, is commonly used to process a SF solution into evenly distributed silk fibers. Normally, the preparation process contains four steps. First, to improve the viscosity and spinnability of the SF solution, an appropriate amount of polyethylene oxide (PEO), which has a high viscosity, is added into the solution and slowly stirred to form a homogenous silk/PEO mixture solution; otherwise, it is difficult to form uniform and continuous silk fibers during the electrospinning process. The ratio of SF to PEO affects the electrospinning effect to a great extent and changes depending on the concentration of the SF solution^73,74^. Second, a proper amount of the silk/PEO solution is drawn up into a syringe with a needle and then spouted onto a collector to form a textile of silk/PEO fibers by electrospinning technology. It is worth noting that the positive and negative poles of a high voltage supply are connected to the needle and the collector, respectively, and there are three crucial parameters, i.e., the flow rate of the silk/PEO solution, electric potential, and distance between the needle tip and the collector, that greatly affect the spun silk/PEO fibers, including the diameter and shape. Third, the obtained textile of silk/PEO fibers is processed with posttreatment, such as water vapor annealing or alcohol (i.e., methanol or ethanol) annealing. These two annealing processes are common methods for inducing β-sheet crystallization of SF. More β-sheet crystals correspond to better mechanical strength, better mechanical toughness, and slower degradation rate^43,45,75^. Finally, the post-treated textile of silk/PEO fibers is immersed in DI water and shaken overnight by a reciprocating shaker until the PEO is completely removed. Figure 3a illustrates a schematic diagram of the electrospinning process of silk fibers, and an advantage is that this method can be used to prepare a three-dimensional woven structure by continuously and uniformly sprinkling sodium chloride (NaCl) particles on a rotating column collector during electrospinning^76^. More commonly, a flat collector is used to collect the electrospun fibers to form a thin textile, as shown in Fig. 3b^77^. In addition to electrospinning technology, in recent years, 3D printing technology has also been introduced by Kaplan et al. to prepare regenerated silk fibers, which can be further stacked into a 3D structure, as shown in Fig. 3c^78^. It is worth noting that 3D printing technology still suffers from many issues in the preparation of silk fibers, such as rapid curing of SF and shape retention of the printed patterns, and moreover, the regenerated silk fibers prepared by 3D printing technology usually have a diameter of more than tens of microns (in contrast to tens of nanometers to several microns for electrospinning).

### Preparation of silk films

**Fig. 4:**
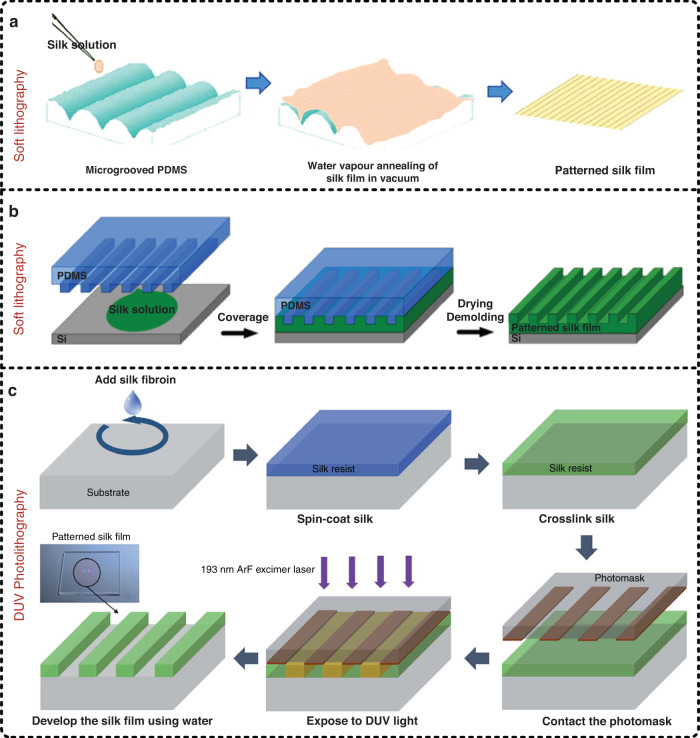
Preparation methods of silk films. **a**, **b** Process flowchart for preparing silk films by using soft lithography^79,81^. Compared to the method shown in **a**, the method shown in **b** can prevent the possible damage to the patterned silk films caused during the transfer process, which is achieved by directly applying a silk fibroin solution onto the destination substrate and subsequently covering it with a patterned mold (e.g., a layer of patterned PDMS). Reproduced with permission from RSC (2017) and Springer (2015). **c** Process flowchart for preparing silk films by applying deep ultraviolet (DUV) photolithography^82^. Reproduced with permission from RSC (2016)

Flexible electronic devices based on film materials are the most common due to their properties of excellent flexibility, ultralight weight, and controllable transmission. Moreover, preparation from solution to film usually shows the advantages of ease of preparation and low cost, and SF is no exception. Figure 4 summarizes the most commonly used preparation methods of silk films. Generally, the preparation method of a silk film with a smooth surface is divided into three steps^43^. First, a SF solution is directly dispersed on top of the surface of a flat substrate such as a plastic Petri dish via a spin coating process. Second, to prevent denaturation of SF, a room temperature (i.e., 25 °C) environment is usually employed to dry SF. It is worth mentioning that a 45 °C environment was used to dry SF in our previous work, which could shorten the drying time, and no visible negative change in optical properties was observed^58^. Third, the dried silk film is immersed in an alcohol solution (i.e., methanol or ethanol) or a water vapor environment to induce crystallization and obtain better mechanical properties as well as the desired degradation rate. In addition, the thickness of the prepared silk film can be controlled by changing the concentration of the SF solution, such as concentrating it for thicker films or diluting it for thinner films. However, a patterned silk film has more application scenarios than a single smooth silk film. Figure 4a illustrates a preparation method of a patterned silk film by pattern transfer (also called soft lithography)^79^. The only difference from the method for preparing a smooth silk film is that the SF solution is dispensed onto a patterned substrate surface (e.g., patterned PDMS). After drying, the obtained silk film has a patterned surface morphology that is complementary to the pattern of the substrate^80^. The method shown in Fig. 4b involves direct application of a SF solution to the top of the destination substrate and subsequent covering of the SF solution with a layer of patterned PDMS for pattern transfer, avoiding possible damage to the patterned silk film during the peeling operation process^81^. In addition, Park et al. reported that SF can be used as a green photoresist for deep ultraviolet (DUV) photolithography, and they also showed some images of patterned silk films prepared by using DUV photolithography. A schematic diagram of the corresponding preparation process is shown in Fig. 4c^82^. Moreover, some other works have also reported patterned silk films prepared by using DUV photolithography^83,84^.

### Preparation of silk sponges

**Fig. 5:**
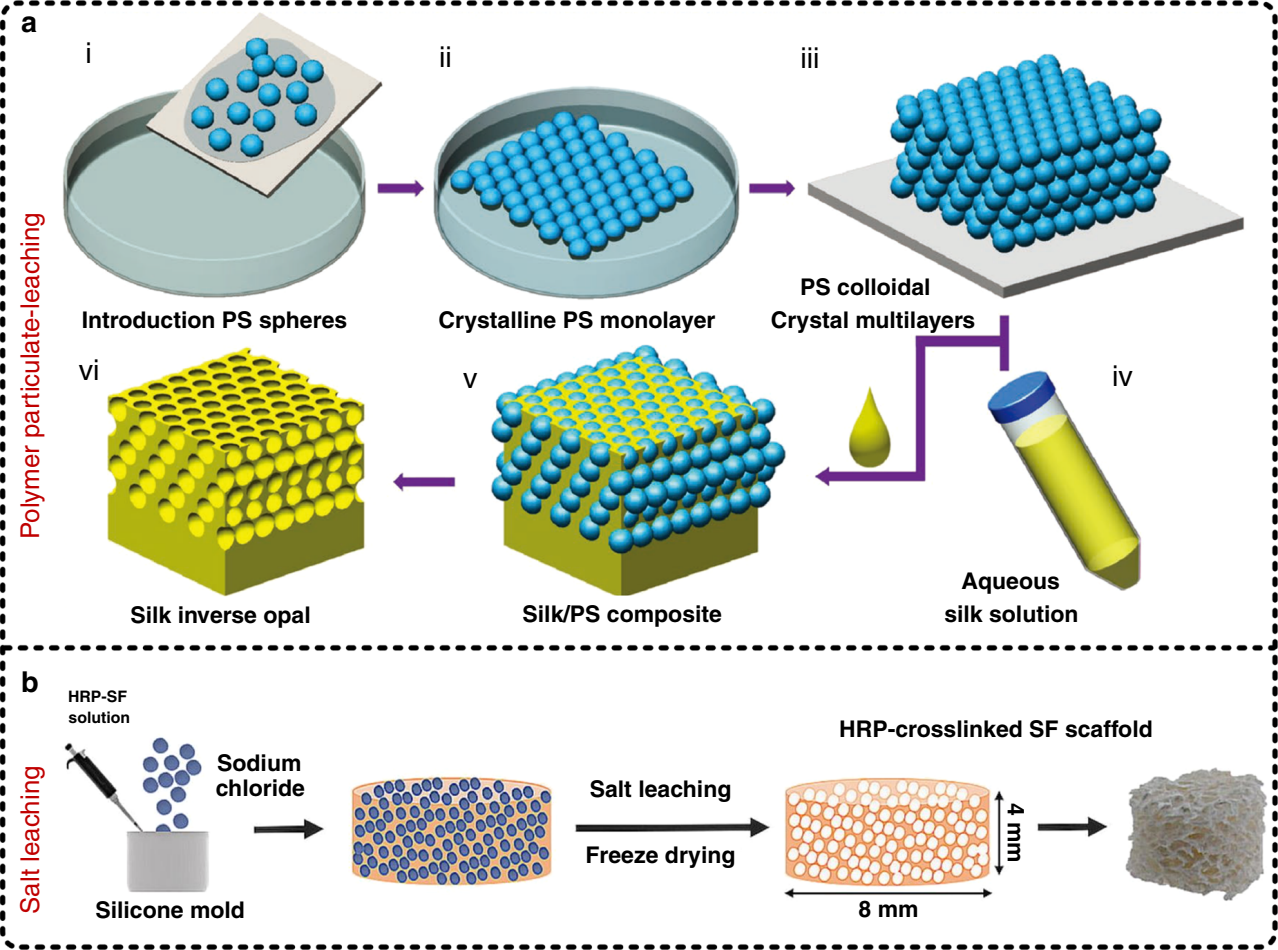
Preparation methods of silk sponges. **a** Process flowchart for preparing silk sponges by employing polymer particulate leaching^94^. Reproduced with permission from Wiley (2017). **b** Process flowchart for preparing silk sponges by using salt leaching. It is worth mentioning that because salt particulates can be partially dissolved into the solvent of the silk fibroin solution, the size of the 3D porous structure obtained via salt leaching is slightly smaller than the size of the salt particulates, and on the other hand, the method of polymer particulate leaching usually requires the introduction of harmful organic solvents to remove polymer particulates, which may negatively affect the biological properties of silk fibroin^95^. Reproduced with permission from Elsevier (2018)

With the help of the large specific surface area, internal crosslinking, and controllable pore size, porous structures are widely applied to enhance the flexibility and performance of electronic devices^85,86^. More interestingly, in the past two decades, some porous biomaterials have made great contributions in biomedicine, e.g., in preparing artificial bones and repairing soft tissues, and moreover, porous SF sponges are particularly prominent as 3D scaffolds due to the large superiority in biocompatibility and controllable biodegradability of SF^87–89^. Several common methods have been introduced in the preparation of porous silk sponges, such as freeze drying^90^, gas foaming^91,92^, and particulate leaching (including salt leaching and polymer particulate leaching)^93,94^. Among them, particulate leaching has the benefits of a simple process, easy control of the pore size, and low equipment requirements. Typically, the preparation of porous silk sponges through particulate leaching contains four main steps. First, particulates, such as polystyrene (PS)^94^, NaCl^95^, and sucrose^93^, with the required diameter are prepared according to the pore size requirements and deposited into a container. If necessary, two sieves with the upper limit (i.e., the maximum pore size) and lower limit (i.e., the minimum pore size) of the required pore size are placed above and below, respectively, to screen particulates. Second, a SF solution is slowly poured into the container onto the top of the particulates and solidified at room temperature. In some cases, the container should be gently rotated to make the particulates distribute evenly. It is worth mentioning that the sequence of the first two steps is not strict and can be adjusted according to the particulates used. Third, the silk/particulate mixture material is immersed in the corresponding solvent, e.g., DI water for NaCl and sucrose and toluene for PS, and slightly stirred until the particulates inside the mixture material are completely removed. During this process, to ensure the removal effect, the solvent needs to be changed five to six times. Eventually, the obtained pure porous silk sponge is moved to room temperature conditions for drying and storage. If the solvent for dissolving particulates in the previous step is an organic solvent, then the porous silk sponge needs to be rinsed in DI water for 24 h before drying. After drying, if necessary, an annealing process can be used to process the dried silk sponge to obtain the desired degradation rate, which is a crucial parameter for in vivo applications. Figure 5a illustrates the schematic process of preparing a 3D porous silk sponge by using PS particulates, which is used to achieve a controllable photonic lattice by changing the diameter of PS particulates^94^. The method shown in Fig. 5b involves the use of NaCl particulates to complete the preparation of a horseradish peroxidase-crosslinked SF scaffold, which is useful for cartilage regeneration^95^. It is worth noting that the pore size of the 3D porous structure obtained by salt leaching (e.g., NaCl) is slightly smaller than the size of the salt particulates used because the salt can be partially dissolved by the solvent of the SF solution during the solidification process. Moreover, polymer particulate leaching (e.g., PS) can well control the pore size, but there is still the defect that harmful organic solvents are usually used to remove particulates.

### Preparation of silk hydrogels

**Fig. 6:**
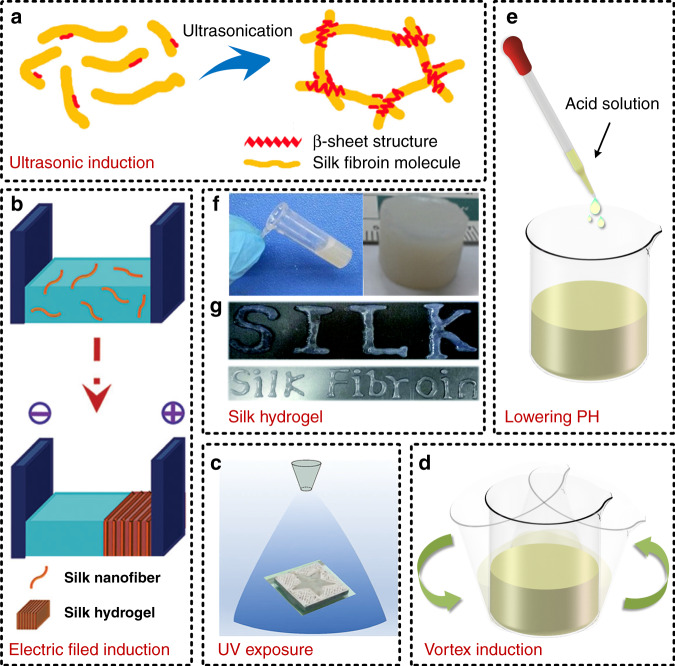
Preparation methods of silk hydrogels. Silk hydrogels that can hold large amounts of water are attractive for implantable applications. The intermolecular and intramolecular interactions of macromolecular protein chains inside silk fibroin make these molecular chains physically crosslinked, resulting in the formation of a silk hydrogel. Some methods are usually implemented to accelerate the gelation process, such as (**a**) ultrasonic induction^106^, (**b**) electric field induction^107^, (**c**) ultraviolet (UV) exposure^108^, (**d**) vortex induction, and (**e**) pH lowering. Reproduced with permission from Springer Nature (2017) and Wiley (2016) and (2017). **f**, **g** Photographs of some samples of silk hydrogels^100,109^. Reproduced with permission from ACS (2016) and RSC (2017)

Silk hydrogels have been demonstrated to have a porous structure similar to that of silk sponges, and they are the intermediate product in some preparation processes for silk sponges^92,95^. However, silk hydrogels are considered to be more suitable for drug delivery and tissue engineering as well as cell culture because of their unique property of holding large amounts of water^96,97^. The mechanism of the gelation effect is that the intermolecular and intramolecular interactions among macromolecular protein chains inside SF, including hydrogen bonds and hydrophobic interactions, make these molecular chains physically crosslinked^98,99^. Therefore, even under normal conditions, a SF solution also has the gelation effect, but it is not used to prepare silk hydrogels due to the large time consumption and low quality. When external factors, such as ultrasonication^100^, an electric field^101^, ultraviolet (UV) irradiation^102^, a rotating water flow^103^, and acids^99,104^, are applied to a SF solution, they advance the interactions among macromolecular protein chains, resulting in rapid physical crosslinking of these chains; thus, a silk hydrogel is obtained. Additionally, these external factors have been shown to facilitate the transition from random coils to β-sheet crystals, which makes the obtained silk hydrogels have better mechanical properties^105^. Figure 6 illustrates schematic diagrams of some commonly used methods for preparing silk hydrogels, including ultrasonic induction^106^, electric field induction^107^, UV exposure^108^, vortex induction, and pH lowering, as well as some photographs of silk hydrogels^100,109^. Among them, mechanical treatment methods, including ultrasonic induction and vortex induction, may destroy the macromolecular chain structure of SF, and the preparation method of pH lowering affects the biocompatibility of SF to a certain extent; thus, a subsequent process needs to be implemented to adjust the pH. The methods of electric field induction and pH lowering can produce silk hydrogels with high viscosity^43^, and another benefit of electric field induction is its reversibility, which means that when the polarity of the power source is changed, the silk hydrogel disappears from the previous positive electrode and forms at the new positive electrode, which was proven by Kaplan et al.^101^. It is worth mentioning that to prevent drying of the prepared silk hydrogel, it needs to be tightly sealed or stored in an environment with high humidity. In addition, compared with other silk-based materials, such as silk films and silk textiles, silk hydrogels have worse mechanical properties. In view of this issue, Su et al. utilized an alcohol posttreatment method to induce the prepared hydrogel to form the crosslinked β-sheet structure again to obtain a dual-network silk hydrogel, which presented good elasticity and mechanical strength at the same time^110^.

## Silk fibroin serving as fundamental components

### Wearable devices

The excellent mechanical properties (i.e., flexibility and stretchability), optical properties (i.e., transparency), biological properties (i.e., biocompatibility), and variety of material forms (i.e., fibers, films, sponges, and hydrogels) make SF valuable or even ideal as substrates for wearable applications^111–113^, and the state of the regenerated solution that is suitable for doping other functional materials further broadens its application range^114,115^. In the last 10 years, a substantial number of flexible and wearable devices based on various forms of SF have been reported, including flexible electrodes, flexible sensors, flexible power supply devices, electronic skins, etc.^116,117^.

**Fig. 7:**
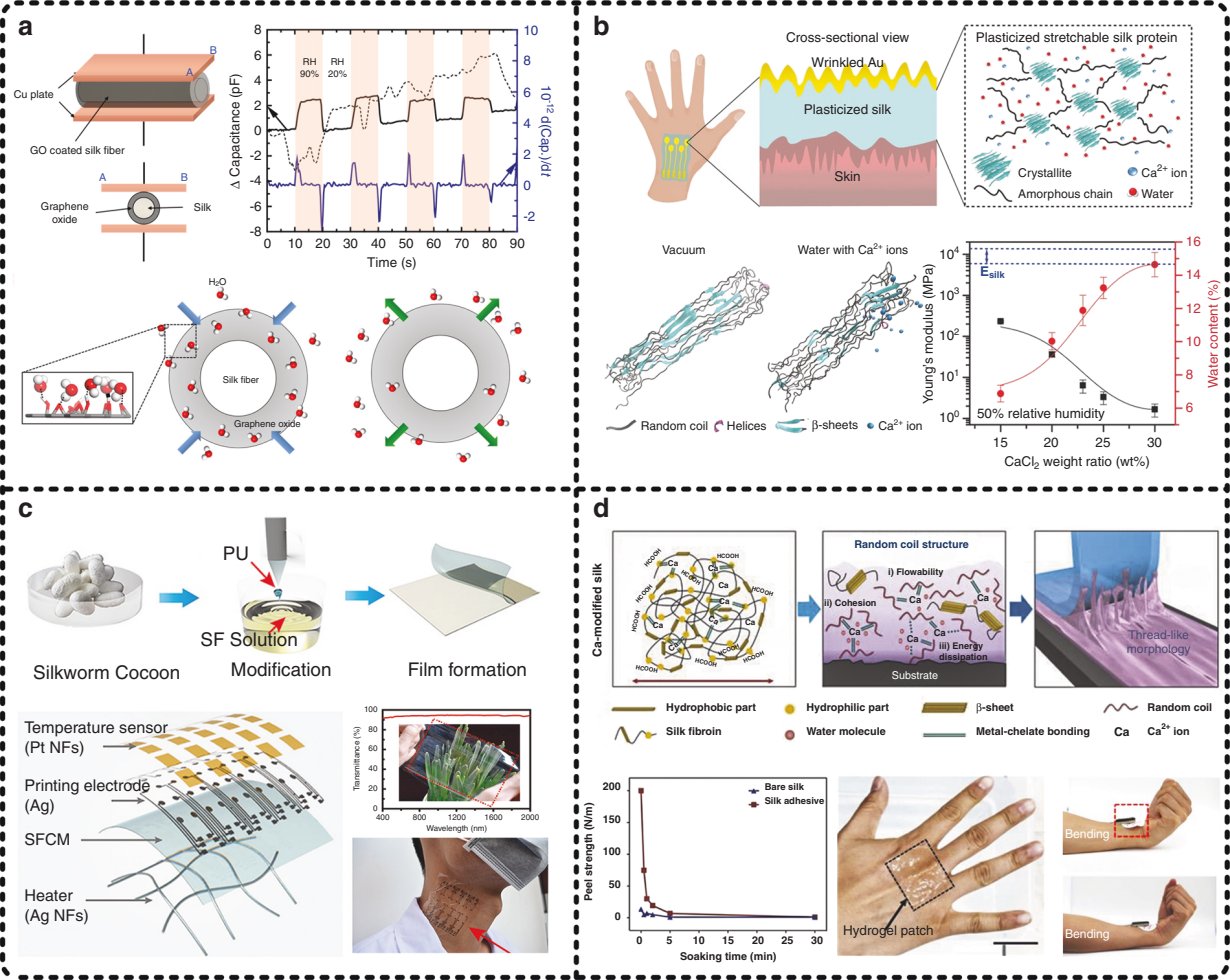
Silk fibroin serves as fundamental components for wearable electronic devices. **a** Han et al. reported a silk fiber-based humidity sensor in which a silk fiber served as a flexible supporting structure and graphene oxide (GO) worked as a functional sensing material^118^. Reproduced with permission from MDPI AG (2017). **b** Chen et al. developed a plasticized silk film through the introduction of calcium chloride (CaCl_2_) and ambient hydration^119^. Reproduced with permission from Wiley (2018). **c** Huang et al. proposed a stretchable and heat-resistant silk fibroin composite membrane (SFCM) to fabricate electronic skins^53^. Reproduced with permission from Wiley (2020). **d** To increase the adhesion of flexible substrates, Seo et al. developed a Ca-modified silk hydrogel that presented excellent stickiness^120^. Reproduced with permission from Wiley (2018)

Figure 7 shows four representative published works, which are selected to explain more details of SF-based materials, including silk fibers, silk films, and silk hydrogels, serving as the fundamental components of wearable electronics. Han et al. developed a humidity sensor in which a silk fiber served as a substrate and graphene oxide (GO) worked as a functional sensing material, as shown in Fig. 7a^118^. The attractive highlight of this work is that GO was attracted to the silk fiber via an electrostatic force, unlike the conventional integration methods of using adhesive intermediates. The mechanism of this method is that GO is negatively charged due to the attached oxygen functional groups, and the silk fiber is positively charged after it is contacted with another material that has a higher ability to obtain electrons; thus, there exists an electrostatic force between GO and the silk fiber. More interestingly, this electrostatic force can be adjusted by selecting a suitable material to contact the silk fiber with. In this work, GO endowed the device with functions, and the introduction of the silk fiber made the device have excellent flexibility.

Compared to silk fibers, SF in film form attracts more attention because it is more convenient to prepare functional components on it. However, silk films prepared from a sole SF solution have almost no stretchability, making them unable to meet the complex shape varieties in human epidermal applications; thus, further development is limited. To address this issue, Chen et al. from Nanyang Technological University developed a plasticized silk film with remarkable stretchability (more than 400%) through the addition of calcium chloride (CaCl_2_) and ambient hydration, as shown in Fig. 7b^119^. Based on combined experimental results and molecular dynamics simulations, they concluded that water can reduce the number of hydrogen bonds and increase the interchain energy, thereby weakening the overall mechanical properties of SF in terms of stiffness and hardness. Meanwhile, Ca^2+^ ions greatly affect the secondary structures of SF, and actually, they mainly lead to an increase in the content of α-helices and random coils that have high extensibility. Therefore, with the help of water and Ca^2+^ ions, the reported plasticized silk film had a lower Young’s modulus and better stretchability. Moreover, in this work, Chen et al. prepared a 40 nm Au film on the plasticized silk film via vacuum deposition to realize a flexible electrode, which presented a low initial sheet resistance of 7 Ω sq^−1^ and a low relative resistance change (*R*/*R*_0_) of 2.45 after being stretched by 40%, so it is expected to be used in on-skin applications. The stretchability of this Au film was achieved by a posttreatment process of ambient hydration to make the Au film produce wrinkled structures.

Electronic skins are considered to be the ultimate form of on-skin electronics, the substrates of which are usually flexible films. Silk films are an ideal material for electronic skins due to their remarkable biocompatibility, superior mechanical properties, and even high transmittance. Huang et al. reported a polyurethane-doped stretchable and heat-resistant SF composite membrane (SFCM) serving as the substrate of electronic skins, as shown in Fig. 7c^53^. It has been reported that SFCMs can be stretched by more than 200% and endure high temperatures over 160 °C; in parallel, they have a high transmittance of over 90%. SFCMs were deposited with silver (Ag) membranes as electrodes, and the two sides of the SFCMs were subsequently processed with a Ag nanofiber (NF) network and a platinum (Pt) NF network, separately, to introduce functions including heating and temperature sensing. It is worth mentioning that no immune response was observed after applying the prepared electronic skin to the human arm for 20 days. Therefore, the excellent mechanical properties, multiple functions, and expected inflammation-free response of the electronic skin developed in this work indicate the feasibility of silk films in the construction of electronic skins.

At present, the development of wearable electronic devices, including electronic skins, is facing many challenges, one of which is strong conformal attachment between the device and the biological surface. Similar to the work shown in Fig. 7b, Seo et al. developed a Ca-modified silk hydrogel as a biocompatible and conformal adhesive suitable for epidermal electronics, as shown in Fig. 7d^120^. The corresponding mechanism for forming viscoelasticity relates to the following three points. First, the presence of Ca^2+^ ions disrupts the crystalline structure during the drying process, so the Ca-modified silk hydrogel is rich in random coils. Second, the metal chelation among random coils provides proper cohesion and causes a large energy dissipation at the interface as an external force is applied. Third, the Ca^2+^ ions inside the Ca-modified silk hydrogel can capture water molecules from the ambient environment. In addition, four different epidermal electronics were well attached to human skin through this viscoelastic silk hydrogel and worked normally, proving its feasibility in on-skin applications.

### Implantable devices

**Fig. 8:**
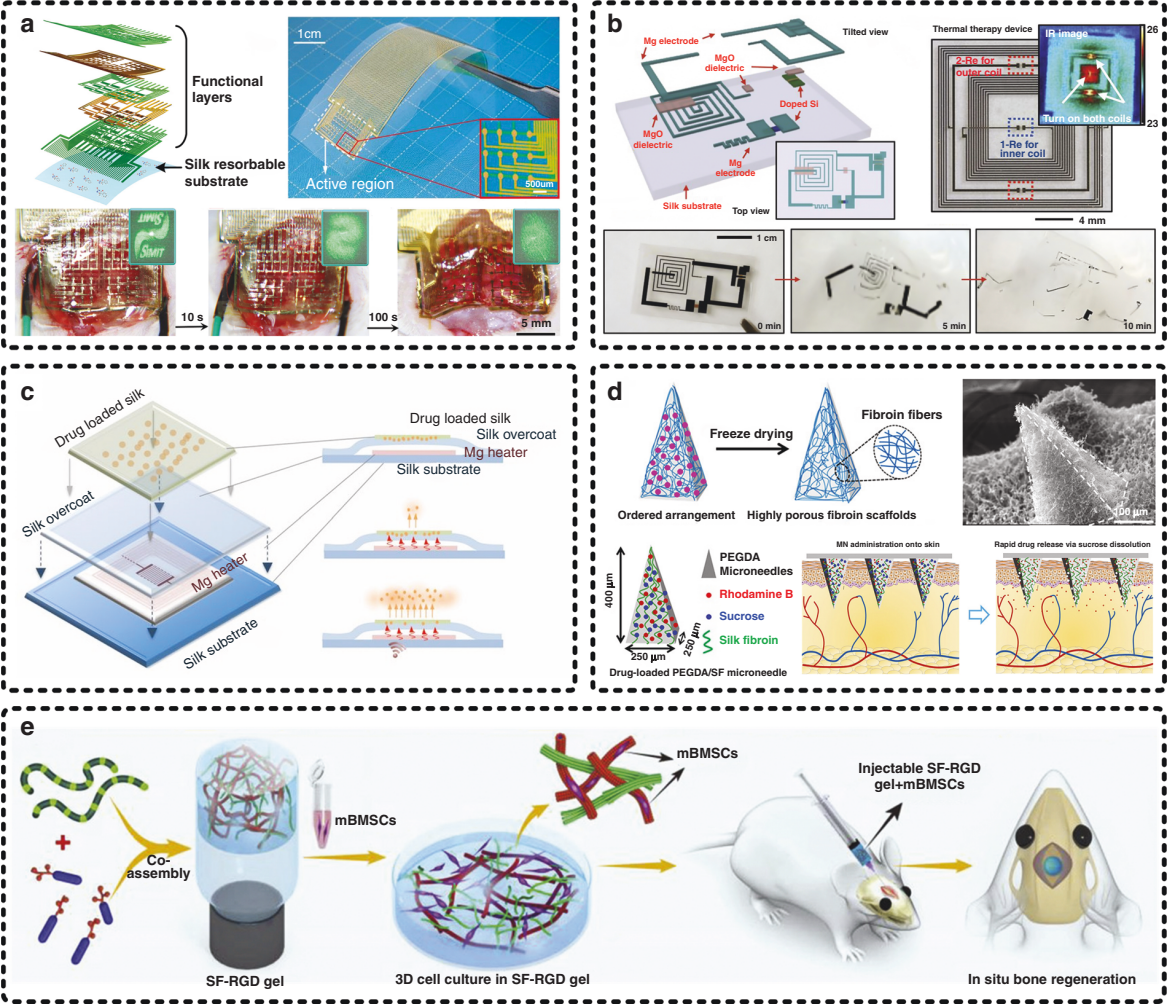
Silk fibroin serves as fundamental components for implantable electronic devices. **a** A silk-enabled conformal multifunctional bioelectronic device was successfully tightly integrated onto the surface of a rat brain by using a silk film as a temporary intermediate medium^129^. Reproduced with permission from Wiley (2019). **b** A silk film-based implantable transient electronic device for wireless thermal therapy and **c** silk film-based controlled drug release technology were codeveloped by Omenetto et al. from Tufts University and Rogers et al. from Northwestern University^54,55^. Reproduced with permission from AAAS (2012) and PNAS (2014). **d** A silk sponge-based implantable microneedle array for minimally invasive drug delivery was proposed by Gao et al.^49^. Reproduced with permission from ACS (2019). **e** A functionalized silk hydrogel for bone defect repair was achieved by introducing a small peptide gelator (e.g., NapFFRGD)^131^. Reproduced with permission from Wiley (2018)

In addition to the above superiorities that are favorable for constituting flexible and wearable devices, SF also has programmable biodegradability and water solubility. Therefore, it is extremely applicable as the fundamental components of implantable devices involving diverse action forms, such as substrates, encapsulation materials, and scaffolds, and in various application directions, such as conformal integration on organ surfaces, wireless therapy, drug delivery, and tissue engineering^121–127^. SF has the characteristics of rapid water dissolution. A silk film can dissolve in DI water within 30 s, and the water solubility of SF can be adjusted by a posttreatment process to change the crystallinity (e.g., alcohol annealing and water vapor annealing)^43,47^, which is important for achieving controllability of the degradation rate of implantable electronics. Huang et al. analyzed the effect of a SF overcoat on the dissolution rate of implantable transient electronic devices and confirmed that the dissolution time of SF-based implantable transient electronic devices strongly depends on the electrode materials used, electrode thickness, and crystallinity of the SF overcoat through modeling analysis and experiments^128^. Figure 8 shows five typical applications of SF-based materials (e.g., silk films, silk sponges, and silk hydrogels), which are selected to exhibit more details. In the recent decade, biological transfer that enables conformal biointegration of biological tissues and flexible electronic devices has aroused some interest. The controllable elasticity, biodegradability, and water solubility imparted by the unique molecular structure of SF make it have great potential for implementing biological transfer. Shi et al. achieved silk-enabled conformal integration in which a multifunctional bioelectronic device was intimately attached to the rat brain surface by using a silk film as a temporary intermediate medium, as shown in Fig. 8a^129^. The temporary silk film was gradually dissolved by applying saline. In parallel, the ultrathin multifunctional bioelectronic device was tightly attached to the surface of the biological tissue (i.e., rat brain) with the help of van der Waals forces and capillary forces. Conformal biointegration allows implantable bioelectronic devices to work better on organ surfaces, such as collecting more accurate electrocorticogram signals.

The more attractive application field of SF in implantable devices is biomedicine due to its extraordinary biocompatibility and controllable biodegradability. Omenetto et al. and Rogers et al. jointly developed an implantable transient electronic device for wireless thermal therapy, as shown in Fig. 8b^55^. In their work, an integrated circuit composed of silicon (Si), silicon dioxide (SiO_2_), magnesium (Mg), and magnesium oxide (MgO) was prepared on a silk film by transfer printing and physical vapor deposition to form a functional electronic device. Among these components, the silk film was easily dissolved in phosphate-buffered saline (PBS), the osmotic pressure and ion concentration of which match those of the human body. The prepared Mg and MgO with a thickness of over 100 nm were experimentally proven to be completely soluble in PBS at room temperature within a dozen hours, and the Si and SiO_2_ over 100 nm could be dissolved in PBS at 37 °C within several weeks. Meanwhile, the implanted device could work normally before the silk film was dissolved, such as receiving wireless power transmission to generate heat for thermal therapy. Similarly, they also jointly developed controllable drug release technology based on a bioabsorbable silk film-based electronic device, as shown in Fig. 8c^54^. In brief, drugs were mixed in a SF solution, and then, a silk film loaded with drugs was obtained through a film preparation process. As featured in the introduction section, the content of β-sheet crystals, which is related to the exposure time in the annealing process, greatly affects the degradation rate of SF; therefore, the drug release rate is adjustable by changing the exposure time in the annealing process. Furthermore, the drug-loaded silk film was integrated with a bioabsorbable Mg heater to accelerate diffusion through wireless driving. In short, silk film-based bioresorbable transient electronic devices have great potential for implantable wireless therapy and drug release due to the controllability of the degradation rate and the avoidance of secondary surgeries.

Figure 8d exhibits an implantable microneedle array based on a silk sponge for minimally invasive drug delivery^49^. The tunable pore size from a few microns to hundreds of microns can bring many benefits in practical biomedical applications, such as adjusting the amount of the loaded drug. In this work, the prepared silk sponge was applied to spontaneously uptake the drug-loaded liquid prepolymer through the capillary force instead of directly mixing the drugs into the SF solution, as in Fig. 8c. Moreover, polyethylene glycol diacrylate (PEGDA) was introduced to provide strong mechanical strength to the microneedle array, and various amounts of sucrose were added to PEGDA to control the drug release kinetics. In addition, SF-based materials, in particular silk hydrogels that can hold large amounts of water, are some of the most promising candidates for tissue engineering^130^. Figure 8e demonstrates a silk hydrogel functionalized for bone defect repair by introducing a small peptide gelator (e.g., NapFFRGD) via cooperative molecular self-assembly^131^. The introduction of the peptide gelator provided better gelation properties of the silk hydrogel, i.e., shortening of the gelation time and improvement of the mechanical strength, and promoted functionalization of the silk hydrogel with cell-adhesive motifs (e.g., RGD), which is beneficial to the interaction between cells and integrin. In this work, the functionalized silk hydrogel was implemented to cultivate mouse bone marrow-derived mesenchymal stem cells and implanted into a mouse with calvarial bone defects, and the experimental results demonstrated the feasibility of using the functionalized silk hydrogel to induce osteogenesis and boost bone regeneration.

## Silk fibroin serving as functional components

### Silk fibroin-based energy harvesters

As briefly described in the “Introduction” section, the further development of flexible electronic devices is confronting many challenges, one of which is a sustainable power supply. A promising candidate to solve this issue is to harvest energy (energies), including thermal energy, mechanical energy, and solar energy, from the human body and surrounding environment and convert it (them) into electricity. Recently, an emerging approach of mechanical energy harvesting technology based on the coupling of the triboelectric effect and electrostatic induction, named the triboelectric nanogenerator (TENG), has attracted much attention due to its excellent characteristics of sustainability, high-output performance, and diverse material selection^132–134^. The working mechanism of TENGs is that when two materials come into contact with each other, charge transfer occurs between them due to their difference in polarity; therefore, a current is generated in the external circuit^133,135^. The polarity of materials determines the ability to gain or lose electrons. Almost all materials we know have been studied, and their abilities to gain or lose electrons have been summarized into a triboelectric series^136^. Interestingly, SF occupies a top-level positive position in the triboelectric series and has a strong ability to lose electrons; therefore, many materials that easily gain electrons can be selected to constitute triboelectric pairs with SF to form high-performance TENGs. Moreover, benefiting from the excellent biological properties and programmable mechanical properties, SF has also been comprehensively studied for constructing biocompatible TENGs. So far the published papers have mainly focused on silk fiber-based TENGs and silk film-based TENGs^137–144^.

**Fig. 9:**
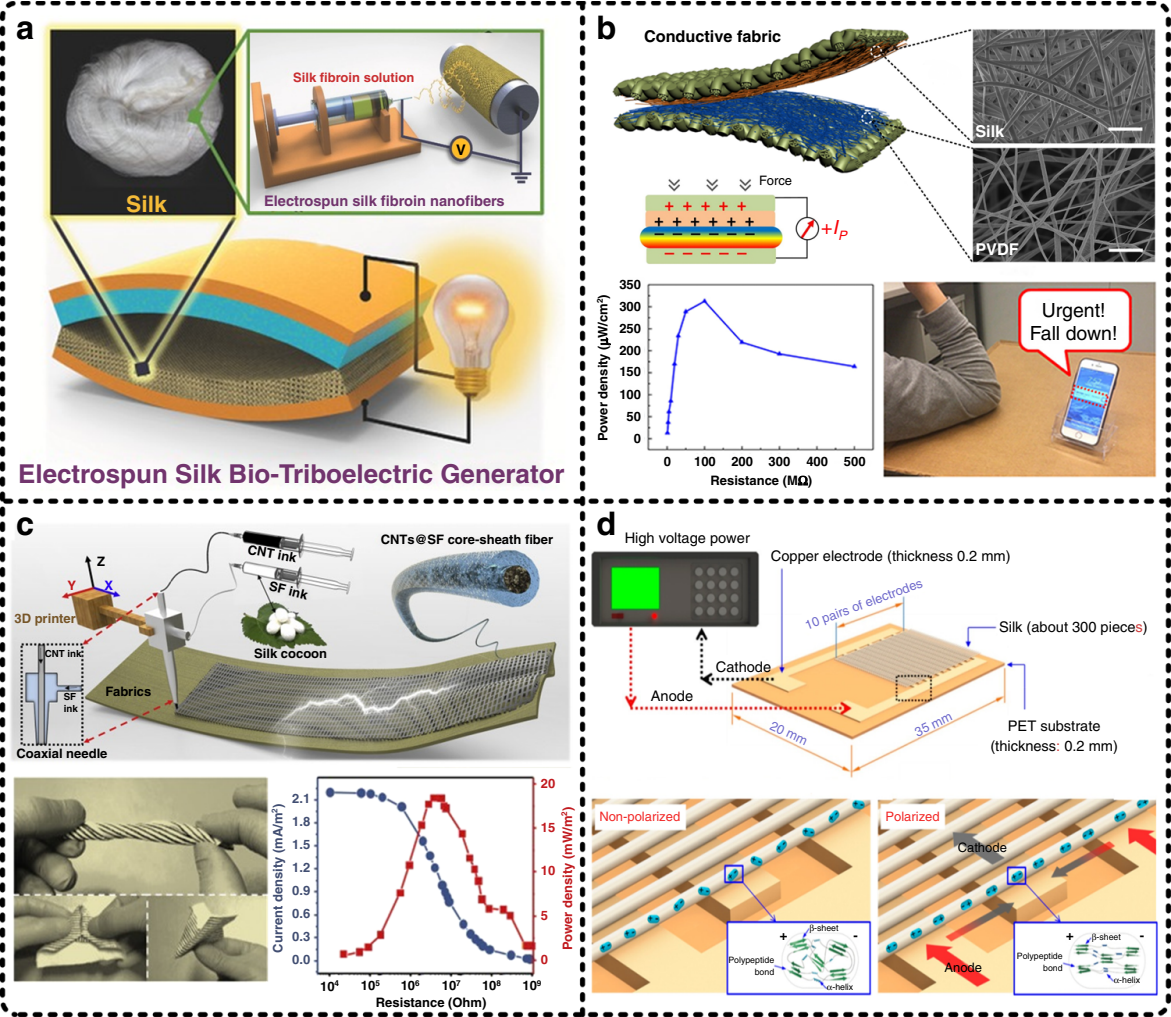
Silk fibers work as active functional materials for the construction of energy harvesters. **a** In 2016, Kim et al. reported a regenerated silk nanofiber-based triboelectric nanogenerator (TENG)^145^. Reproduced with permission from Wiley (2016). **b** In our previous work published in 2018, we developed an all-fiber piezoelectric-enhanced TENG, which was made of electrospun silk nanofibers and poly(vinylidene fluoride) (PVDF) nanofibers^48^. Reproduced with permission from Elsevier (2018). **c** In 2019, Zhang et al. applied a 3D-printed coaxial composite fiber composed of carbon nanotubes and silk fibroin (CNTs@SF) to fabricate TENGs^146^. Reproduced with permission from Elsevier (2019). **d** In addition to silk fiber-based TENGs, silk fiber-based piezoelectric nanogenerators (PENGs) were also reported, such as the repolarized natural spider silk fiber-based PENG proposed by Pan et al.^150^. Reproduced with permission from ACS (2018)

The characteristics of air permeability and large surface-to-volume ratio of fiber networks make silk fibers receive considerable attention for constituting TENGs. Figure 9a–c illustrates three representative works on silk fiber-based TENGs to go into the merits in detail. In 2016, regenerated silk NFs prepared by electrospinning to construct an ecofriendly bio-TENG were first introduced by Kim et al., as shown in Fig. 9a^145^. In this study, a PI film was selected to constitute a triboelectric pair with a silk fiber membrane. As a result, the bio-TENG showed a high-output performance with a triboelectric surface charge density of 1.86 μC/m^2^ and a maximum instantaneous power density of 4.3 mW/m^2^ at a load resistance of 5 MΩ, and the durability and reliability of the bio-TENG were proven to be excellent owing to the outstanding mechanical properties of the regenerated silk NF networks. In 2018, we developed an all-fiber piezoelectric-enhanced TENG by preparing electrospun silk NFs and poly(vinylidene fluoride) (PVDF) NFs onto conductive fabrics, as shown in Fig. 9b^48^. With the help of the large surface-to-volume ratio of both NF networks, the strong ability of SF to lose electrons, and the piezoelectric enhancement of PVDF, the all-fiber TENG demonstrated a very high-output performance with a maximum power density of 3100 mW/m^2^. In parallel, this novel all-fiber configuration endowed the textile-based piezoelectric-enhanced TENG with excellent flexibility and expected air permeability, which make it very beneficial for integration with clothes and achieving health monitoring (e.g., urgent falls), indicating its great potential for integrated wearable applications. In 2019, Zhang et al. reported a core-sheath fiber-based TENG, as shown in Fig. 9c^146^. In their work, 3D printing technology was applied to prepare a coaxial composite fiber of carbon nanotubes and SF (CNTs@SF), where CNTs served as a conductive core and SF served as a dielectric sheath. When the other dielectric material of the triboelectric pair was polyethylene terephthalate (PET), the maximum power density of the core-sheath fiber-based TENG was measured as 18 mW/m^2^. The scientific merits of this work are that the core-sheath fiber-based configuration achieved high integration of functional materials and electrodes, and 3D printing technology endowed the textile-based device with customizable patterns.

In addition to being a dielectric material for TENGs, SF was also found to have piezoelectric properties, which were first reported by Harvey in 1939 and were first measured quantitatively by Fukada in 1956^147,148^. However, it was only in recent years when the piezoelectric properties of SF truly attracted the attention of researchers and were applied to build piezoelectric energy harvesters, also named piezoelectric nanogenerators. The piezoelectric properties of SF derive from the noncentrosymmetric crystal structure of β-glycine and γ-glycine inside SF^149^. Figure 9d illustrates a silk fiber-based piezoelectric energy harvester, which was reported by Pan et al.^150^. In this work, a repolarization process was implemented to enhance the piezoelectric properties, resulting in an increase in the piezoelectric output from 13.4 to 40.7 mV with a vibration of 4 Hz. The mechanism is that repolarization makes the secondary structures of silk fibers rearrange more orderly, resulting in a reduction in internal neutralization. The piezoelectric energy harvester made of the repolarized silk fibers showed a maximum output power of 59.5 pW when the load resistance was 8.2 MΩ. More works on silk fiber-based piezoelectric energy harvesters have also been reported^151,152^, and the reported maximum load output power density has reached 45.6 mW/m^2^ ^56^, which demonstrates the great potential of SF for biocompatible piezoelectric energy harvesting.

**Fig. 10:**
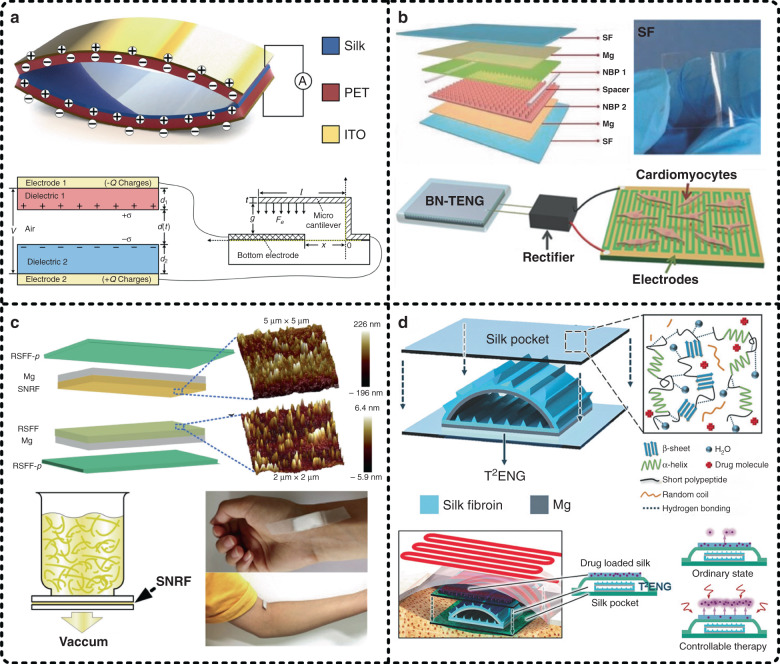
Silk films work as active functional materials for the construction of energy harvesters. **a** A silk film-based TENG was applied to power a mechanical microcantilever in our previous work, which is actually the first work that employs silk fibroin as a functional dielectric layer to constitute a triboelectric pair^47^. Reproduced with permission from Elsevier (2016). **b** A bioabsorbable TENG composed of natural materials (i.e., silk fibroin, cellulose, chitin, egg white, rice paper, etc.) was integrated into a self-powered stimulation system for dysfunctional cardiomyocyte clusters^153^. Reproduced with permission from Wiley (2018). **c** A double-silk TENG consisting of a regenerative silk fibroin film (RSFF) and a silk nanoribbon film (SNRF) to retain the superior biological properties of silk fibroin was developed^154^. Reproduced with permission from Elsevier (2020). **d** An automatic therapy system for epilepsy was achieved by integrating a silk film-based TENG, a drug-loaded silk film, a wireless emitter, and a heating unit^155^. Reproduced with permission from Wiley (2018)

Additionally, silk film-based TENGs have the superiority of easy processing, and high-output performance can also be achieved by processing microstructures on the material surface as well as applying an oxygen plasma process to improve the roughness and interfacial energy of silk films. Four representative silk film-based TENGs are illustrated in Fig. 10. As shown in Fig. 10a, we proposed a transparent TENG based on a silk film, which is actually the first work introducing SF as a functional dielectric layer into the construction of TENGs^47^. In this work, the triboelectric pair of the TENG was composed of SF and PET, which were processed with an oxygen plasma process to achieve a high-output performance up to 1936 mW/m^2^ (load resistance of 40 MΩ). As a potential application prototype, the TENG was applied to power a mechanical microcantilever that is required by autonomous sensor networks. As shown in Fig. 10b, Jiang et al. proposed fully bioabsorbable TENGs based on natural materials, such as SF, cellulose, chitin, egg white, and rice paper^153^. In this work, SF served simultaneously as an encapsulation material and one of the functional dielectric materials for energy harvesting, and biodegradable Mg worked as electrodes. To demonstrate the application potential and practicality, the bioabsorbable natural material-based TENG was integrated into a self-powered stimulation system to generate electric stimulation, accelerating the beating rates of dysfunctional cardiomyocyte clusters and improving the consistency of cell contraction.

In all the above reports, SF is just one of the materials for constituting triboelectric pairs. Recently, Niu et al. developed a biocompatible TENG in which both materials of the triboelectric pair and even encapsulation materials were silk-based materials, as illustrated in Fig. 10c^154^. In brief, one of the dielectric materials was the commonly used regenerative silk fibroin film (RSFF); the other dielectric material was a silk nanoribbon film (SNRF), which was prepared by successive implementation of a 2,2,6,6-tetramethyl-piperidine-l-oxyl radical/sodium bromide (NaBr)/sodium hypochlorite (NaClO) oxidation system and an ultrasonic treatment process as well as a centrifugation process to obtain a silk nanoribbon (SNR) from natural silk, and the SNR was subsequently vacuum filtered to form a film. The electrodes were made of Mg. Moreover, encapsulation layers were made of posttreatment RSFF (RSFF-*p*), which had an adjustable lifetime. Since the degree of crystallinity and surface morphology of the RSFF and SNRF were different, their abilities to gain/lose electrons were also discrepant. As a result, the double-silk TENG achieved a high-output performance of 86.7 mW/m^2^ at a load resistance of 100 MΩ. In summary, the double-silk configuration provided TENGs with very excellent biocompatibility, biodegradability, and controllable lifetime, making them have outstanding potential as wearable power sources, especially implantable power sources. Additionally, Zhang et al. also reported an implantable TENG made of SF and Mg, as shown in Fig. 10d^155^. The difference is that Mg served as electrodes and one of the functional dielectric materials for energy harvesting at the same time in this work. The maximum output power density of this implantable TENG was 38.5 mW/m^2^ when the load resistance was 100 MΩ. More interestingly, in this work, the implantable TENG was integrated with automatic therapy for epilepsy by loading drugs into the silk encapsulation. When an epileptic symptom occurs, a heating unit is triggered by the TENG because of the generated abnormal output signals, resulting in the loaded drugs being quickly released. In addition to silk film-based triboelectric energy harvesters, silk film-based piezoelectric energy harvesters have also been reported^156,157^. In summary, we are convinced that these reported advanced energy harvesters based on SF have confirmed its value and potential, and SF-based materials will continue to receive attention in biocompatible energy harvesting.

### Silk fibroin-based sensors

**Fig. 11:**
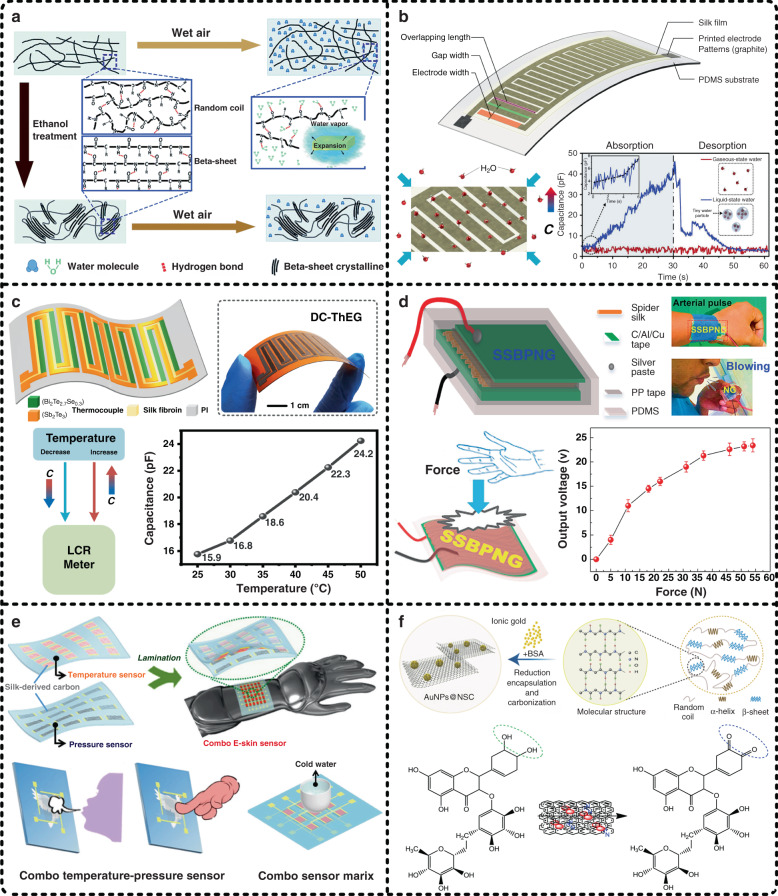
Silk fibroin is used as a functional material to develop flexible sensors. **a** Li et al. reported a silk membrane-based humidity sensor based on the optical properties of silk fibroin^164^. Reproduced with permission from RCS (2017). In our previous work, we developed two kinds of silk film-based sensors, **b** one of which was used to sense the existence of liquid-state water in air^57^ and **c** the other one of which applied silk fibroin as a temperature-dependent material to detect temperature^58^. Reproduced with permission from Elsevier (2019) and Springer Nature (2020). **d** With the help of the inherent piezoelectric properties of spider silk, Karan et al. proposed a spider silk-based bio-piezoelectric nanogenerator (SSBPNG), which was also applicable as a pressure sensor^56^. Reproduced with permission from Elsevier (2018). **e** Wang et al. developed an all silk-derived dual-mode electronic skin in which integrated pressure and temperature sensing functions were achieved^59^. Reproduced with permission from ACS (2017). **f** Patil et al. reported a silk-derived flexible electrochemical sensor for the detection of rutin^175^. Reproduced with permission from Elsevier (2019)

For another key issue in the further development of flexible electronics, i.e., multifunctional integration, SF also presents great potential for solving it due to its diverse functional properties, such as humidity sensitivity, temperature dependence, and pressure sensitivity. In recent years, SF has received rapidly increasing attention as a functional material for developing flexible sensors, including humidity sensors^158,159^, temperature sensors^58^, pressure sensors^156,160^, electrochemical sensors^161,162^, and airflow sensors^163^. Figure 11 illustrates six representative contributions of silk fibrin-based sensors to exhibit the corresponding details. Li et al. developed a SF-based humidity sensor with the help of the quantitative relationship between the reflection peak of a silk membrane and humidity^164^. The authors prepared silk membranes with various thicknesses ranging from more than 100 nanometers to hundreds of nanometers by tuning the spin coating rate. Taking into account the entire uniformity of the samples, the silk membranes obtained at a spin coating rate of 3000 rpm were selected to analyze the optical response of SF to humidity in this work. When the silk membranes absorb water molecules in wet environments, the protein chains inside SF change, and the volume of the silk membranes expands, resulting in apparent redshifts of the reflectance peaks; conversely, blueshifts of the silk membrane reflectance peaks can be observed in dry environments; the degree of reflectance peak shifts depends on the content of water molecules in the environment. Therefore, by comparing the color of silk membranes, the corresponding humidity can be determined. Moreover, the authors proved that the ethanol-treated silk membranes showed a smaller color change because the high crystallinity of SF could prevent water infiltration. Figure 11a illustrates the mechanism of the humidity responses of the original and ethanol-treated silk membranes. In addition to applying optical mechanisms^165,166^, capacitance-dependent SF-based humidity sensors have also been reported^167^. Furthermore, it is worth mentioning that there are two states of water in air, liquid-state water and gaseous-state water, both of which affect the humidity. Traditional humidity sensors respond to water in both states and thus cannot distinguish these two states. In our previous work, we prepared a layer of a 3 μm silk film atop PDMS to form a SF-based water molecule sensor for distinguishing the existing states of water in air according to the tremendous difference in the capacitive response to water in different states, as shown in Fig. 11b^57^. Gaseous-state water in air is water vapor, and liquid-state water in air represents tiny water particles that have balanced gravity and buoyancy. In other words, liquid-state water in air contains many more water molecules, rendering a significantly more intense capacitive response of the SF-based water molecule sensor, which can be proven by the difference between the Fourier transform infrared spectrum of an original silk film and that of a liquid-water treated silk film. Moreover, the proposed SF-based water molecule sensor was successfully demonstrated to monitor human respiratory states, showing great potential for health monitoring.

In another work, we used SF as an active functional component for temperature sensing, which was coupled with a double-chain thermoelectric generator to realize the integration of sensing and energy harvesting, as shown in Fig. 11c^58^. The mechanism of temperature sensing can be mainly described as two points: an increase in temperature intensifies the molecular motion of SF, and the intensity of molecular motion affects the dielectric constant of SF, which determines the capacitance of SF-based temperature sensors. In this work, the capacitance of the developed SF-based temperature sensor presented a good linear dependence on temperature, indicating an attractive feasibility for temperature sensing.

Pressure sensing devices are one of the most widely used sensors, application fields of which include industrial processing, medical healthcare, and logistics management, etc. Pressure sensors that apply SF as active functional components mainly include two mechanisms, i.e., the piezoelectric effect^156^ and the resistive principle^168^. Figure 11d illustrates a spider silk-based bio-piezoelectric nanogenerator (SSBPNG) realized by utilizing the innate piezoelectric properties of spider silk, which was used as a pressure senor to detect physiological signals^56^. In this work, spider silk fibers with diameters from 8 to 11 μm collected from a spider were adopted to form the piezoelectric layer, which was covered by a layer of PDMS to ensure that there was no short between the two electrodes (i.e., C/Al/Cu tape). The entire prepared SSBPNG was encapsulated by PDMS to protect it from external environmental hazards (e.g., mechanical stress, humidity, and temperature), and polypropylene tape was set between the device and PDMS to make it free from any triboelectric effect. As a result, the SSBPNG presented a maximum power density of ~45.6 mW/m^2^, and the function of sensing biological behavior and motions, e.g., arterial pulse, swallowing, coughing, blowing, and writing, was successfully achieved by using the SSBPNG as a pressure sensor. Similarly, Joseph et al. reported an ultrasmooth silk thin film-based pressure sensor based on the inherent piezoelectric properties of SF, which exhibited an average sensitivity of 3.26 mV/kPa^169^. Additionally, silk-derived carbon fibers were found to have good conductivity, revealing their attractive potential as resistive sensors^170,171^. As shown in Fig. 11e, Zhang et al. developed a silk-derived dual-mode electronic skin integrating pressure sensing and temperature sensing^59^. To obtain conductive silk carbon fiber membranes, a thermal treatment process was implemented to carbonize the electrospun silk fibers into conductive pseudographitic pyroproteins, which possess a randomly oriented localized graphitic structure. The authors experimentally demonstrated that the continuous carbonized silk fibers possessed good temperature dependence due to rapid heat transfer, and the fractured carbonized silk fibers were sensitive to pressure owing to the large number of contact points. These two silk-derived carbon fiber membranes were processed into two separate sensor arrays and then integrated into a dual-mode electronic skin, which was implemented to detect exhalation, finger pressing, and object location.

In addition, SF has also been used as a chemically dopable functional material to prepare flexible electrochemical sensors because it is an excellent precursor for chemical doping^172–174^. Figure 11f illustrates a silk-derived carbon/metal composite material-based flexible sensor for the chemical detection of rutin^175^. The authors applied the high reduction ability of bovine serum albumin molecules to reduce ionic Au and then mixed Au nanoparticles into a SF solution. Subsequently, the mixed solution was subjected to freeze drying and carbonized in a nitrogen atmosphere to eventually form a conductive Au nanoparticle/nitrogen-doped silk carbon (AuNPs@NSC) composite material, which showed strong electrocatalytic activity in rutin sensing. Furthermore, the prepared AuNPs@NSC-based sensor was demonstrated to be effective and accurate; thus, it proved the potential to extend the electrochemical applications of SF to the flexible sensing field.

### Other silk fibroin-based flexible devices

**Fig. 12:**
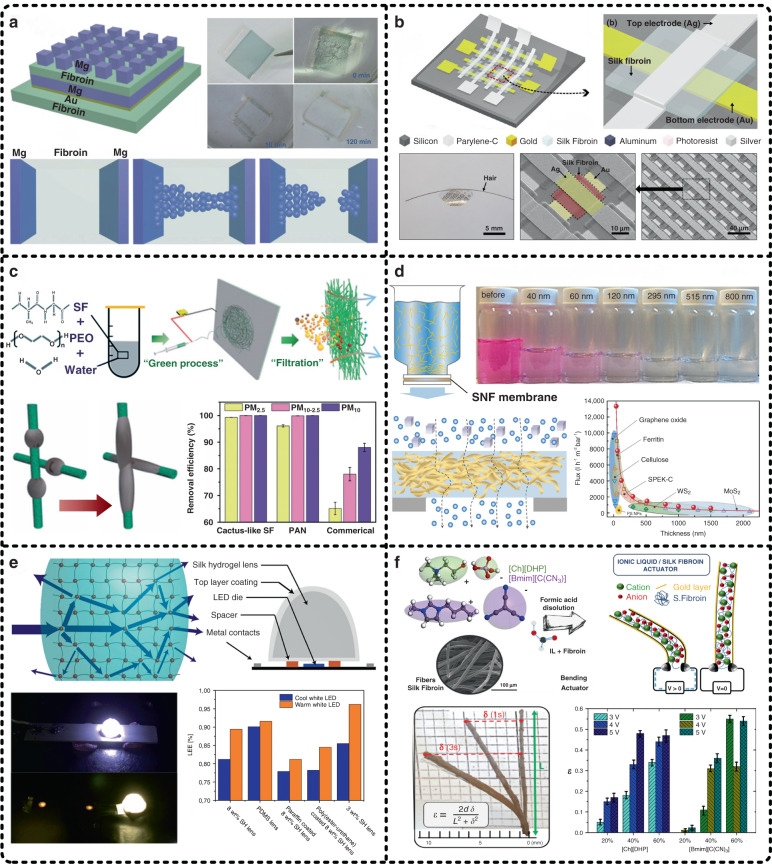
Silk fibroin is implemented as a functional material for the construction of other flexible devices. **a** A resistive switching memory device^197^ and **b** a resistive switching memory array based on silk fibroin were realized by utilizing the excellent dielectric properties of silk fibroin^61^. Reproduced with permission from Wiley (2016) and (2020). **c** A high-performance silk nanofiber-based air filter was proposed with the help of the hydrophilic behavior of silk nanofiber membranes^199^. Reproduced with permission from RSC (2018). **d** A highly ordered multilayer membrane made of silk nanofibrils (SNFs) and hydroxyapatite (HAP) nanocrystals was developed for water purification^200^. Reproduced with permission from ACS (2016). **e** A silk hydrogel lens with high light extraction efficiency (LEE) was achieved by optimizing the concentration of a silk fibroin solution and internal crosslinking^62^. Reproduced with permission from Springer Nature (2017). **f** A biological bending actuator was developed by using ion-doped silk fibroin as an active material^204^. Reproduced with permission from ACS (2019)

In addition to energy harvesters and sensors based on SF, other flexible functional devices using SF as an active functional material have also received considerable attention, such as resistive switching memory devices^176–178^, OFETs^179,180^, filters^181–186^, OLEDs^187^, and actuators^188–190^. Resistive switching memory devices that can be switched between a high resistance state (HRS, OFF state) and a low resistance state (LRS, ON state) through a varying electric field are considered promising candidates for next-generation high-performance data memory^191–196^. As depicted in Fig. 12a, Wang et al. utilized the controllable water solubility and excellent biocompatibility as well as outstanding dielectric properties of SF to develop transient resistive switching memory^197^. The resistive switching mechanism can be described as follows: when a positive voltage is applied to the Mg electrodes, a large electric field is generated, which compels the Mg nanoparticles to migrate across the silk film and then form conductive filamentary paths, leading to the LRS; when a negative voltage is applied to the Mg electrodes, a large tunneling gap is formed, and the conductive filamentary paths are broken, resulting in a state change from the LRS to the HRS. Consequently, the proposed SF-based resistive switching devices achieved a resistance ON/OFF ratio of greater than 10^2^ and a retention time of more than 10^4^ s, and moreover, the SF-based transient resistive switching memory can dissolve in DI water or PBS after 2 h. Furthermore, Kook et al. developed a flexible high-density resistive switching array by using UV photolithography, as shown in Fig. 12b^61^. The resistance ON/OFF ratio of this resistive switching array reached 10^9^, which is the highest value among the SF-based resistive switching devices reported thus far.

Nanonetwork-based air filters made from electrospun biomaterials were confirmed to be effective and regarded as a green technology due to the lack of pollutants in the process of preparation and operation^198^. SF has attracted special attention in the research of biological air filters because it not only has excellent biological properties but also possesses a large number of functional groups, including hydroxyl groups, amide bonds, and phenolic hydroxyl groups, that facilitate the capture of various particles or chemicals. As shown in Fig. 12c, a highly effective SF-based air filter was proposed by Gao et al.^199^. With the help of the surface tension of SF and the adhesion between pollutants and silk NFs, this SF-based air filter presented excellent filtration performance. The hydrophilic behavior of silk NF membranes was conducive to the deformation and spreading of the collected pollutants without causing spherical blockages; therefore, the removal efficiency of air filters made of silk NF membranes was higher than that of commercial filters and hydrophobic polyacrylonitrile NF membranes. In addition to air filters, SF has also received attention for water purification applications. Ling et al. reported an ultrathin nanoporous filtration membrane made of silk nanofibrils (SNFs) for the removal of contaminants in water, including dyes, proteins, and other nanoparticles, as illustrated in Fig. 12d^200^. The SNFs were directly exfoliated from natural *Bombyx mori* silk fibers to retain their structure and physical properties, and moreover, ultrathin SNF membranes were ultimately fabricated by vacuum filtration. As a result, the ultrathin SNF membranes (i.e., 40 nm) developed in this work had a pure water flux of 13,000 l h^−1^ m^−^^2^ bar^−1^, which was much higher than that of most commercial ultrathin filtration membranes prepared from polysulfone, poly(ether sulfone), and polyamide. Excellent broad-spectrum separation performance of the SNF membranes for most dyes, proteins, and nanoparticles was confirmed. In addition to size-derived physical filtering, the authors proposed a nanofibril/hydroxyapatite (SNF/HAP) composite membrane to remove metal ions by interacting with metal ions through chelation and ion exchange^201^. The SNF/HAP membranes were proven to have the superiorities of excellent adsorption capacity for the removal of contaminants and low cost.

The remarkable optical transmittance of SF endows it with attractive potential in the optical-electronic device field. As demonstrated in Fig. 12e, Melikov proposed a silk hydrogel (SH) lens for light-emitting diodes (LEDs)^62^. The scattering phenomena inside the SH lens caused by the various local refractive indices between proteins and water broadened the viewing angle. Through experimental comparison, it can be concluded that both the concentration and the degree of crosslinking affect the optical properties of SF, and moreover, the authors proved the high light extraction efficiency of SH lenses, in particular for warm white LEDs. Furthermore, a wax coating approach was investigated to enhance the stability of SH lenses by more than three orders of magnitude.

Beyond these, novel actuators based on flexible materials, especially biomaterials, are gaining increasing interest^202,203^. Figure 12f illustrates a bending actuator composed of ionic liquid (IL)/SF composite films^204^. In this work, 1-butyl-3-methylimidazolium tricyanomethanide ([Bmim][C(CN_3_)]) and choline dihydrogen phosphate ([Ch][DHP]) ILs were used to introduce ions into SF and increase the alternating current conductivity and dielectric constant of SF. When an electric field is applied to an IL/SF composite film, a bending response occurs owing to the respective diffusion displacement of anions and cations to the negative and positive electrodes. The bending response value was defined as *ε* = 2*dδ*/(*L*^2^ + *δ*^2^), where *d* is the thickness of the sample, *L* is the free length of the sample, and *δ* is the maximum displacement of the sample under an applied voltage. As a result, the maximum value of *ε* was calculated to be up to 0.5, and it was confirmed that this value depends on the amount of ions introduced, regardless of the type of IL. This work proved the attractive feasibility of using ion-doped SF as an active material to construct flexible biological actuators.

## Conclusions and outlooks

SF has been demonstrated to possess many superior properties, including remarkable biocompatibility, adjustable biodegradability, and water solubility, excellent optical transmittance, good mechanical robustness, light weight and ease of processing, which render it extremely suitable for the construction of next-generation biocompatible flexible electronic devices. Some preparation technologies, such as electrospinning, 3D printing, spin coating, soft lithography, freeze drying, particulate leaching, and ultrasonic induction, have been introduced to process SF materials in diverse forms, i.e., silk fibers, silk films, silk sponges, and silk hydrogels, for various application areas. Owing to the desired biological properties, SF is widely used as fundamental components, i.e., substrates and encapsulation materials as well as scaffolds, of flexible wearable and implantable electronic devices, such as electronic skins, bioabsorbable electronics, and therapy electronics. In addition, in recent years, flexible electronic devices in which SF serves as a functional material have gained more attention. First, SF was found to have piezoelectric properties and a strong ability to lose electrons; therefore, it was used as a dielectric material to build piezoelectric energy harvesters and triboelectric energy harvesters. Second, owing to the characteristics of SF sensitive to environmental variables, many flexible sensors with SF functional components have been proposed, such as humidity sensors, temperature sensors, pressure sensors, airflow sensors, and electrochemical sensors. Third, some other types of flexible electronic devices using SF as a functional material were also developed, including resistive switching memory devices, OFETs, OLEDs, filters, and actuators, which further expanded the application field of SF. Table 1 summarizes the recent SF-based applications.

**Table 1:** Applications of silk fibroin in the field of flexible electronics

Applications	Types	Characteristics involved	References
Wearable electronics	Substrate, encapsulation	Biocompatibility, biodegradability, water solubility, flexibility, transparency, stretchability	^51–53,111–120^
Implantable electronics	Substrate, encapsulation	Biocompatibility, biodegradability, water solubility, flexibility	^49,54,55,121–131^
Energy harvesters	Functional layer	Strong ability to lose electrons, flexibility, stretchability, biocompatibility	^47,48,56,137–157^
Sensors	Functional layer	Sensitivity to environmental variables, flexibility, stretchability, biocompatibility	^57–59,156–170,173–175^
Biomemristors, OFETs	Substrate, functional layer	Dielectric properties, flexibility, biocompatibility, biodegradability	^61,176–180,197^
Filters	Substrate, functional layer	Flexibility, hydrophilicity, nanonetwork structure	^60,181–186,199,200^
LED lenses	Functional layer, encapsulation	Transparency, internal crosslinking	^62,187^
Actuators	Substrate, functional layer	Dielectric properties, flexibility, stretchability, ion dopability	^188–190,204^

Nevertheless, many challenges still prevent the practical development of SF-based flexible electronic devices. First, although SF-based electronic devices are superior in terms of flexibility and biocompatibility, their performance is still inferior to that of traditional silicon-based electronics; thus, more attempts are required to improve the performance of SF-based flexible electronic devices so that they can actually substitute rigid silicon-based electronics. Second, SF lacks mechanical strength in the state of high water content, and it is brittle in the state of low water content; therefore, the optimal balance state needs to be explored, and new methods are expected to be developed to simultaneously retain the high mechanical strength and excellent flexibility of SF. Third, in general, metal electrodes or polymer conductors need to be introduced into SF-based flexible electronic devices for enhancement of the conductivity, but they weaken the biocompatibility and biodegradability of the whole devices; thus, one of the next research directions could be introducing biocompatible and even bioabsorbable conductive materials into SF-based flexible electronic devices or further studying conductive silk-derived carbon materials. In addition, even though many preparation processes have been introduced to prepare SF in a variety of forms, most of these methods, especially regarding the preparation processes for patterning, fail to meet the requirements of large-scale production. In other words, large-scale preparation processes need to be developed to fabricate patterned SF materials in batches. It is worth mentioning that due to the unique advantages of SF, including compatibility, controllable biodegradability, and water solubility, some researchers have paid attention to all silk flexible electronics, which may be one of the next crucial research directions. In summary, SF has opened up a new road for the flexible electronics field, and although many challenges still need to be overcome on this road, we are convinced that more attempts will be made by researchers to advance the practical development of SF-based electronics in flexible wearable and implantable microsystems.

## Supplementary information

Supplemental Material
