# Supplementary material for: Recent progress in silk fibroin-based flexible electronics
Source: Microsyst Nanoeng. 2021 May 6;7:35. doi: 10.1038/s41378-021-00261-2 (PMC8433308; doi:10.1038/s41378-021-00261-2)

**Supporting Information file**

Recent Progress in Silk Fibroin-Based Flexible Electronics

Dan-Liang Wen^1^, De-Heng Sun^1^, Peng Huang^1^, Wen Huang^1^, Meng Su^2^, Ya Wang^3^, Meng-Di Han^4^, Beomjoon Kim^2^, Juergen Brugger^3^, Hai-Xia Zhang^4^, Xiao-Sheng Zhang^1,*^

^1^School of Electronic Science and Engineering, University of Electronic Science and Technology of China, Chengdu 611731, China

^2^CIRMM, Institute of Industrial Science, The University of Tokyo, 153-8505 Tokyo, Japan

^3^Microsystems Laboratory, École Polytechnique Fédérale de Lausanne (EPFL), 1015 Lausanne, Switzerland

^4^Institute of Microelectronics, Peking University, 100087 Beijing, China

*Corresponding Author: Xiao-Sheng Zhang ([zhangxs@uestc.edu.cn](mailto:zhangxs@uestc.edu.cn))

**Table S1**. Comparison of flexible natural biomaterials mentioned in this review (based on the reviewed references [31-43]).


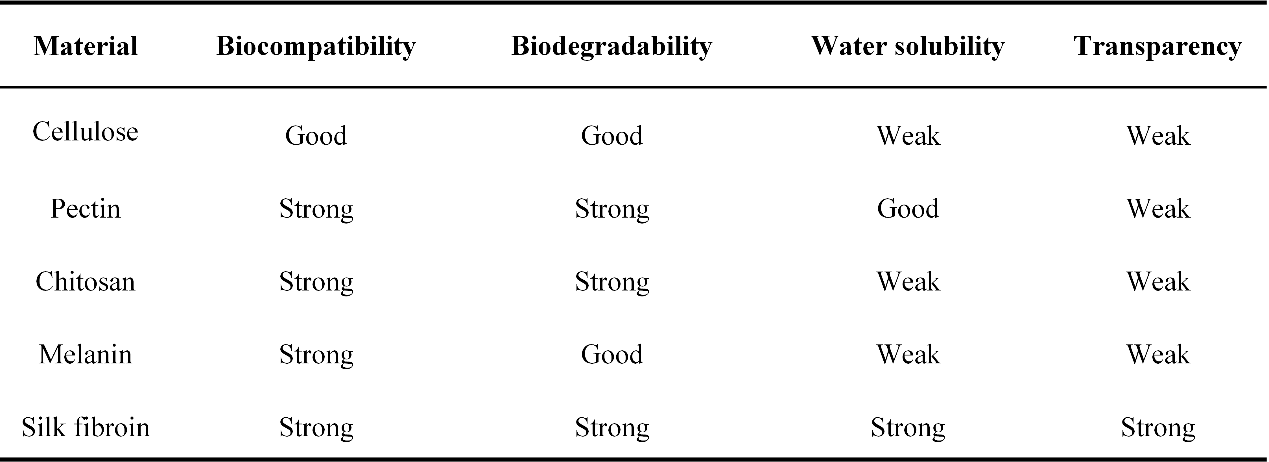

Supplement: Supplementary file 1 — Supplemental Material [file 41378_2021_261_MOESM1_ESM.docx]
